# A modified technique of transanal specimen extraction in the laparoscopic anterior rectal resection for upper rectal or lower sigmoid colon cancer: a retrospective study

**DOI:** 10.1186/s12893-021-01085-7

**Published:** 2021-02-12

**Authors:** Si Yu, Yong Ji, Tedong Luo, Pengjie Xu, Zuojun Zhen, Jianzhong Deng

**Affiliations:** 1 Department of General Surgery, The Second People’s Hospital of Foshan, Foshan, China; 2 Department of General Surgery, The First People’s Hospital of Foshan (Foshan Hospital of Sun Yat-Sen University), Foshan, China

**Keywords:** Transanal specimen extraction, Laparoscopic anterior rectal resection, Colorectal cancer

## Abstract

**Background:**

In recent years, natural orifice specimen extraction surgery (NOSES) has become a field of special interest for colorectal surgeons. Some researchers have reported transanal specimen extraction in the laparoscopic anterior rectal resection, including intersphincteric resection (ISR) and rectal eversion-resection. However, these surgical procedures have certain limitations. Based on the proven expertise in laparoscopic surgery, our center has developed a modified technique of transanal specimen extraction. The aim of this study was to investigate the safety and feasibility of a modified technique of transanal specimen extraction in the laparoscopic anterior rectal resection.

**Methods:**

From January 2011 to January 2014, the patients with upper rectal or lower sigmoid colon cancer who had undergone laparoscopic anterior rectal resection with specimen extraction by a modified transanal technique were enrolled in the observation group, and the patients who had undergone laparoscopic anterior rectal resection with specimen extraction via an abdominal incision by the same surgeons during the same period were enrolled in the control group.

**Results:**

A total of 36 patients were included in the observation group and 128 patients were included in the control group. There were no significant differences (*P* > 0.05) between the two groups in terms of the mean operative time [144 ± 10 min vs. 141 ± 11 min], mean intraoperative blood loss [63 ± 6 ml vs. 61 ± 7 ml], and the mean time to anal exhaust [67 ± 7 h vs. 65 ± 8 h]. However, there were significant differences (*P* < 0.05) between the two groups in terms of the mean postoperative Visual Analogue Scale (VAS) pain scores [3.4 ± 1.1 vs. 4.5 ± 1.2], mean postoperative hospital stay [6.0 ± 1.1 days ± vs. 7.2 ± 1.2 days], and incidence of postoperative complications (4/36 vs. 15/128). Long-term follow-up results showed that there was no significant difference (*P* > 0.05) between the two groups in terms of the 3- or 5-year overall survival.

**Conclusions:**

The modified technique of transanal specimen extraction in the laparoscopic anterior rectal resection fulfilled the principle of no-neoplasm touch technique, with advantages, such as minimal trauma, rapid recovery, and fewer complications. Long-term follow-up results also showed satisfactory oncological outcomes.

## Background

Minimally invasive laparoscopic surgery has been widely used for the treatment of colorectal cancer [1–3]. For upper rectal or lower sigmoid colon cancer, laparoscopic anterior rectal resection with specimen extraction via an abdominal incision has been performed conventionally [4, 5]. However, the scar on the abdomen caused by this procedure may cause dissatisfaction in some patients. In recent years, some researchers have reported transanal specimen extraction in laparoscopic anterior rectal resection [6–8], including intersphincteric resection (ISR) [9–11], and rectal eversion-resection [12, 13]. However these surgical procedures have certain limitations. For example, the rates of complications, such as anastomotic leakage and postoperative anal sphincter incontinence, are relatively high. In addition, they are only applicable to the middle and low rectal cancer, but not to upper rectal and sigmoid colon cancer. Based on the proven expertise in laparoscopic surgery, our center has developed a modified technique of transanal specimen extraction which is suitable for upper rectal and sigmoid colon cancer. We found that the technique was easy to operate and reduced complications such as anastomotic leakage. In this study, we report a retrospective analysis of outcomes in the patients with upper rectal or lower sigmoid colon cancer who had undergone laparoscopic anterior rectal resection with specimen extraction by the modified transanal technique. To further confirm the effect, we included the patients as controls who had undergone laparoscopic anterior rectal resection with specimen extraction via an abdominal incision.

## Methods

### General materials

The data was retrieved from the database in the medical record room of our center. The criteria for case ascertainment include: (1) Adenocarcinoma was confirmed by pathology; (2) The tumor was located in the upper rectum or lower sigmoid colon; (3) The operation was completed under laparoscope; (4) Time was from January 2011 to January 2014.

All of the patients underwent preoperative colonoscopy to obtain pathological diagnosis. The determination of tumor location comes from colonoscopy, MRI or CT. The division of the rectum and sigmoid colon is usually marked by the position of the 15 cm above the anal verge. The maximum transverse diameter of tumors was measured by MRI or CT.

The patients with upper rectal or lower sigmoid colon cancer who had undergone laparoscopic anterior rectal resection with specimen extraction by the modified transanal technique were included in the observation group. The patients who had undergone laparoscopic anterior rectal resection with specimen extraction via an abdominal incision were included in the control group.

All of the patients signed the consent form before the operation. The surgical procedures in the two groups were approved by the Medical Ethics Committee of our Hospital, and they were performed during the same period by the same team of surgeons with extensive experience in laparoscopic surgery.

### Surgical methods

**Fig. 1:**
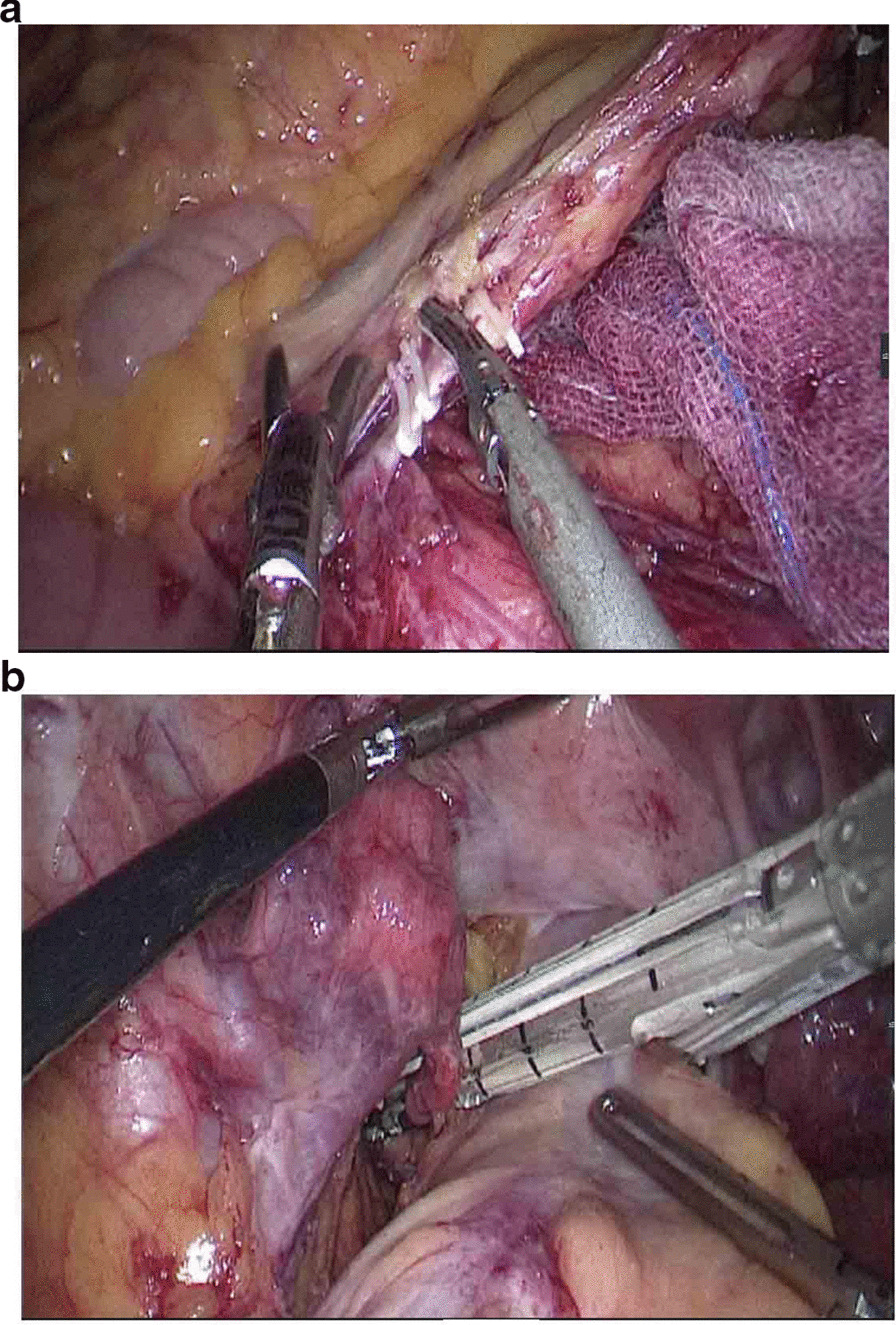
**a** The inferior mesenteric artery was dissected at the root. **b** A linear stapler was used to cut and close the rectum

**Fig. 2:**
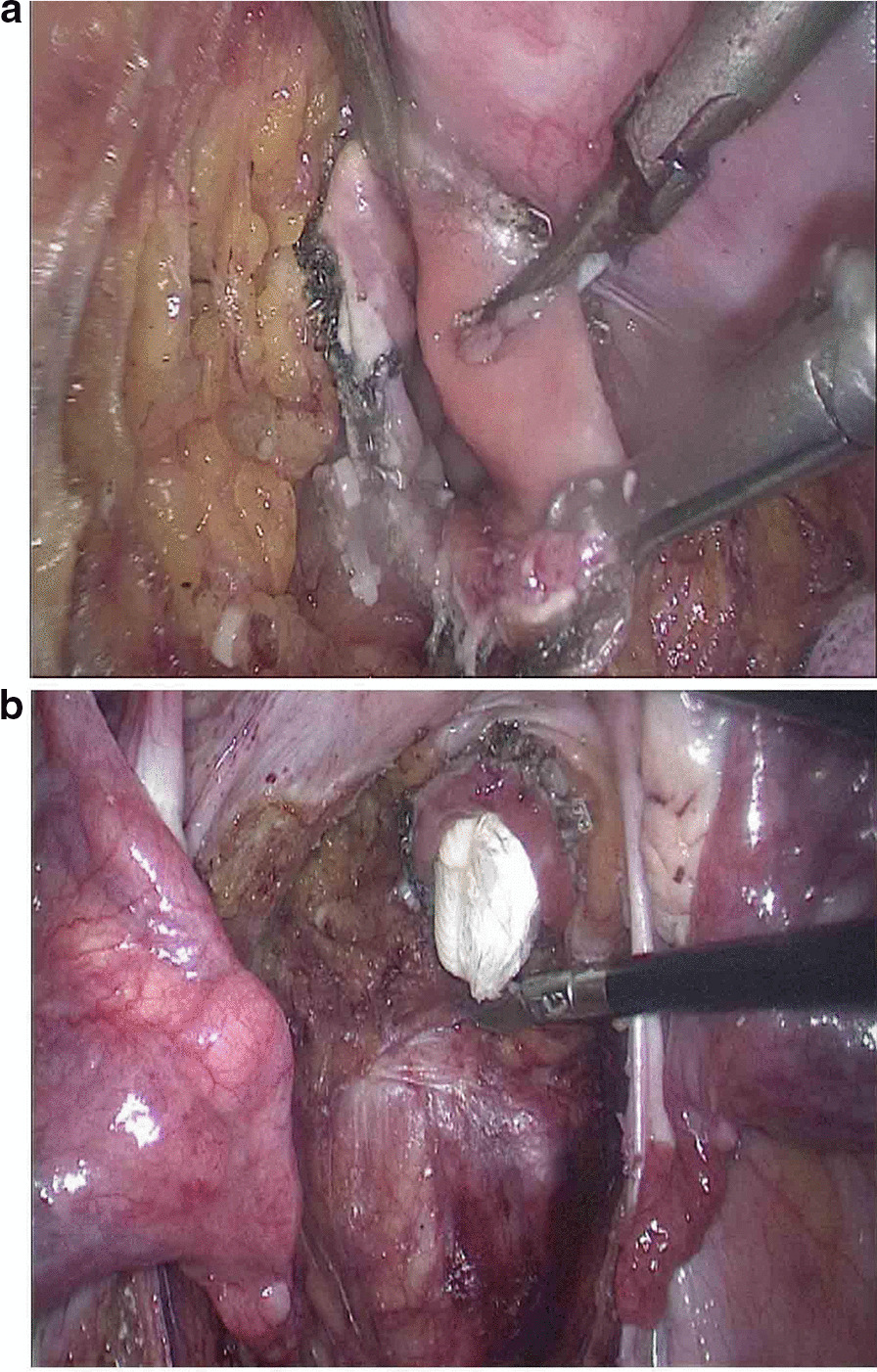
**a** The stump of distal rectum was incised under laparoscopic vision. **b** One end of a sheath-shaped sterile bag about 30–40 cm long was inserted into the pelvic cavity through the anus

**Fig. 3:**
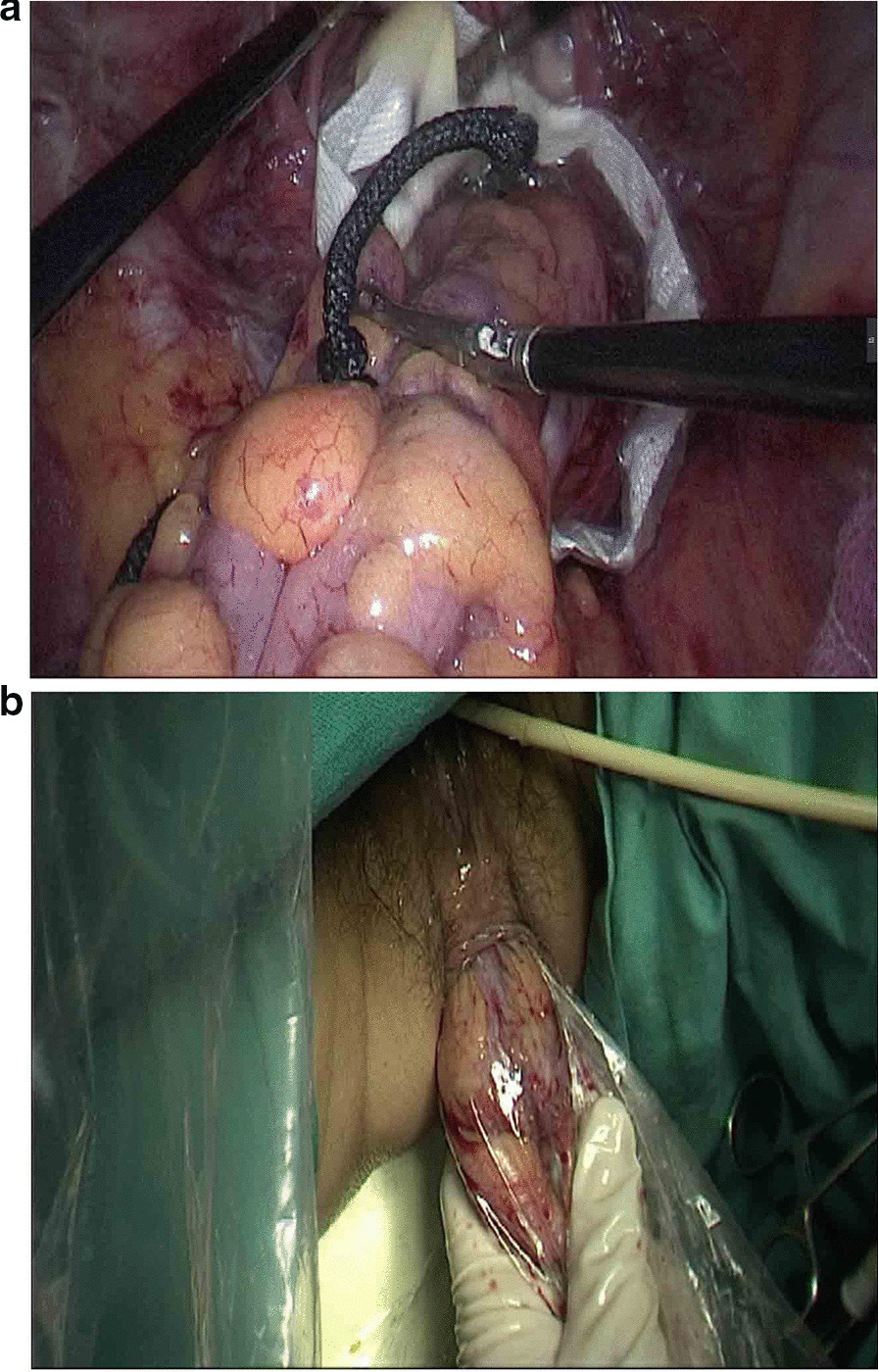
**a** The mobilized and tumor-containing proximal colon segment was placed into the bag. **b** The colon segment and the sterile bag were synchronously pulled out of the anus

**Fig. 4:**
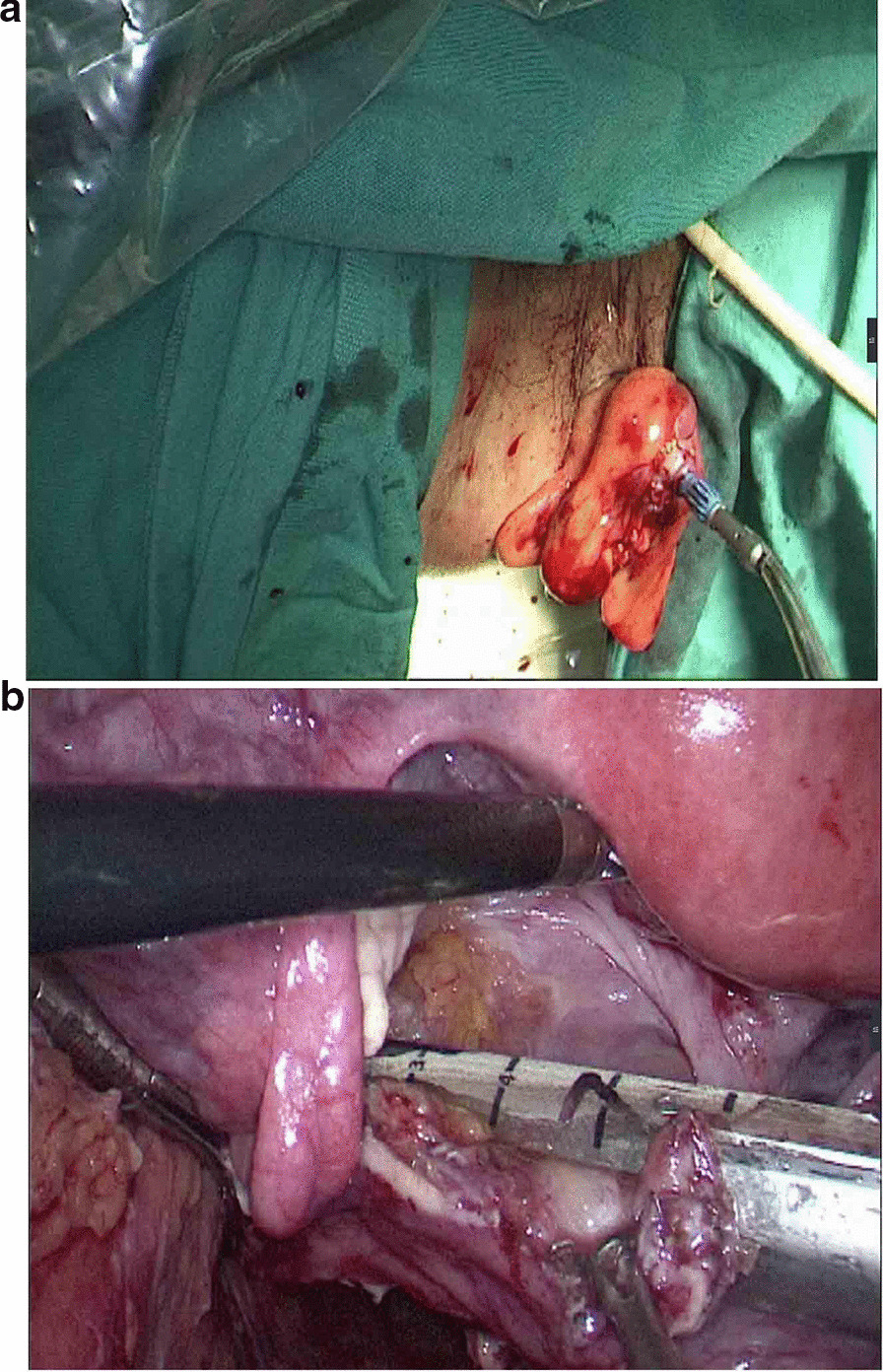
**a** The retained proximal colon was attached with a stapling anvil and placed transanally back to intraperitoneal cavity. **b** A linear stapler was used to close the stump of rectum

**Fig. 5:**
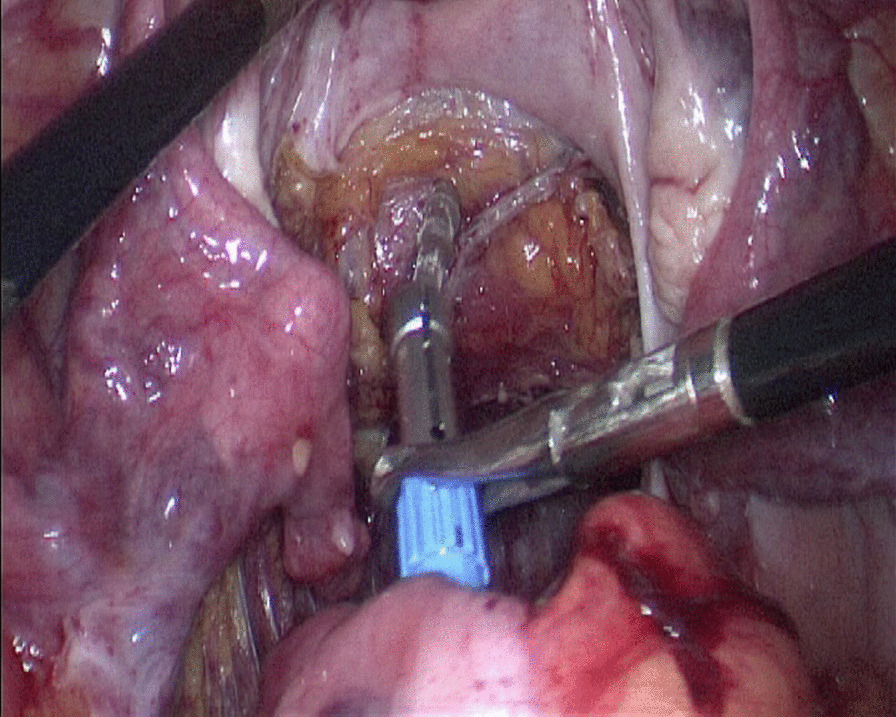
A stapler was inserted through the anus and colorectal anastomosis was completed

The observation group: An indwelling catheter was placed before the operation, and general anesthesia was induced via tracheal intubation. Each patient was placed in a lithotomy position, with the feet higher than the head by 15°–20°, and leaning rightward by 10°–15°. Using a routine five-port technique, a laparoscope was introduced through the umbilical port. The McBurney's point on the right side of the abdomen was used as the primary operative port; a first auxiliary operative port 5 mm in size was made on the right side of the umbilical hole; two auxiliary operative ports were arranged in the left lower quadrant. Routine intraperitoneal exploration was performed, and the feasibility of transanal specimen extraction was evaluated. Through a medial approach, the inferior mesenteric artery was dissected at the root (Fig. 1a). The inferior mesenteric vein was dissected along the inferior margin of the pancreas. The left hemicolon and rectum were separated in the Toldt's gap. The left ureter and reproductive vascular system were protected. The sigmoid mesocolon was trimmed and the marginal vascular arcade was protected. At the distal end 5 cm away from the tumor, the rectum was “naked” and a linear stapler (Echelon 60, Ethicon Endo-Surgery, Cincinnati, USA) was used to cut and close the rectum (Fig. 1b). The distal rectum was irrigated thoroughly with a diluted povidone-iodine solution after perineal re-disinfection. The feasibility of transanal specimen extraction was re-assessed. The stump of the distal rectum was incised under laparoscopic vision (Fig. 2a). A pair of oval forceps was used to insert one end of a sheath-shaped sterile bag about 30–40 cm long into the pelvic cavity through the anus, with the other end staying outside the anus (Fig. 2b). The mobilized and tumor-containing proximal colon segment was placed into the bag (Fig. 3a). Subsequently, the bag opening was tightened and the tumor-containing proximal colon segment was ligated, so that the tumor was completely isolated. A pair of toothed oval forceps was placed in the bag, and it was used to enter the pelvic cavity through the anus, grip the proximal colon segment, and pull the colon segment and the sterile bag synchronously out of the anus through the distal rectum (Fig. 3b). The colon was dissected at the proximal end 10 cm away from the tumor; the retained proximal colon was attached with a stapling anvil and placed transanally back into the intraperitoneal cavity (Fig. 4a). Then a linear stapler was used to close the stump of the rectum (Fig. 4b). Finally, a stapler was inserted through the anus, and colorectal anastomosis was completed (Fig. 5).

The control group: the first half of the procedure was the same as that in the observation group. After the rectum was transected with a cutter stapler, a 5-cm median longitudinal incision was made on the lower abdomen. An incision protective sleeve was used to protect the incision. The proximal colon was pulled out and dissected 10 cm away from the tumor. Specimens were removed; the retained proximal colon was attached with a stapling anvil and placed back into the intraperitoneal cavity. After re-establishment of pneumoperitoneum, a stapler was inserted through the anus, and colorectal anastomosis was completed.

### Comparative indexes and statistical methods

The comparison between the two groups included preoperative data, such as tumor location, tumor differentiation, tumor diameter, etc., and intraoperative and postoperative data, such as operative time, blood loss, time to anal exhaust, etc.

The treatment costs of the patients in two groups were also compared. Treatment costs refers to the total expenditure of patients from hospitalization to discharge, including drug cost, operation cost, medical consumables cost, nursing cost, etc.

Patients were followed up by mail, telephone interview or outpatient clinic questionnaire. Enumeration data are expressed as mean ± SD and compared using the *t*-test. Measurement data were compared using the chi-square test. SPSS v19.0 statistical software was used for data analysis. *P* < 0.05 was considered to be statistically significant.

## Results

A total of 36 patients, including 20 males and 16 females, aged 46–76 years (mean 62 ± 7 years, were included in the observation group. A total of 128 patients, including 70 males and 58 females, aged 46–81 years (mean 61 ± 8 years), were enrolled in the control group. The data in Table 1 show that there were no statistical differences between the two groups in terms of age, sex, body mass index (BMI), tumor position, tumor differentiation, and American anesthesiologist classification (ASA) (P > 0.05), except for tumor diameter (P < 0.05).

**Table 1:** Comparison of preoperative demographic data between the two groups (x^2^ ± s)

	Observation group	Control group	*P* value	*T/X*^*2*^ value
Number of patients	36	128		
Age (years)	62 ± 7	61 ± 8	0.335	0.966
Sex				
M	20	70	0.926^c^	0.009
F	16	58		
BMI (kg/m^2^ )	23.0 ± 1.7	23.4 ± 1.7	0.298	− 1.043
Tumor position				
Lower sigmoid colon	15	60	0.579 ^c^	0.307
Upper rectum	21	68		
Tumor differentiation				
High	6	26	0.856^c^	0.311
Moderate	27	90		
Low	3	12		
Tumor diameter^a^ (cm)	4.2 ± 0.5	4.6 ± 0.6	0.000^b^	− 4.442
ASA classification				
I	8	20	0.632^c^	0.916
II	22	87		
III	6	21		

*BMI* body mass index, *ASA* American Society of anesthesiologist

^a^Maximum transverse diameter of a tumor intestinal segment measured by CT or MRI scan

^b^P < 0.05 is considered statistically significant

^c^Chi-square test

In the two groups, none of the patients required conversion to open laparotomy and the operative mortality was zero. The mean operative time, mean amount of blood loss, mean time to anal exhaust and mean postoperative hospital stay in the observation group were (144 ± 10) min, (62 ± 7) ml, (67 ± 7) h and (6.0 ± 1.1) days, respectively. The patients’ pain degree was assessed on the basis of Visual Analogue Scale (VAS) [14]. In the observation group the patients had a mean postoperative VAS pain score of (3.4 ± 1.1). Four patients had postoperative complications, including anastomotic fistula in two patients, urinary retention in one patient, and pneumonitis in one patient. The average number of harvested lymph nodes was 14.6 (ranging from 12 to 18). According to the seventh edition of UICC TNM Classification of Malignant Tumors, 5 patients were classified as stage I, 19 patients as stage II, and 12 patients as stage III.

In the control group, the mean operative time, mean amount of blood loss, mean time to anal exhaust and mean postoperative hospital stay were (141 ± 11) min, (61 ± 7) ml, (65 ± 8) h and (7.2 ± 1.2) days, respectively. The patients had a mean postoperative VAS pain score of (4.5 ± 1.2). Fifteen patients had postoperative complications, including incisional infection in five patients, anastomotic fistula in six patients, urinary retention in two patients, and pneumonitis in two patients. The average number of harvested lymph nodes was 13.9 (ranging from 12 to 17). According to the seventh edition of UICC TNM Classification of Malignant Tumors, 15 patients were classified as stage I, 77 patients as stage II, and 36 patients as stage III.

Statistical analysis showed that the two groups had no significant difference (*P* > 0.05) in terms of the mean operative time, intraoperative blood loss, and postoperative time to anal exhaust. The observation group had a significantly lower mean postoperative VAS pain score than the control group (P < 0.05), and a shorter mean postoperative hospital stay than the control group (P < 0.05). There was no significant difference (P > 0.05) between the two groups in terms of the overall postoperative complications. None of the patients in the observation group had incisional infection, while five patients in the control group had incisional infection, suggesting that there was a significant difference (P < 0.05) between the two groups in terms of incidence of the incisional infection. The mean treatment cost in the observation group was higher than that in the control group. However, there was no significant difference (P > 0.05) between the two groups in terms of the treatment cost. The data of intraoperative and postoperative conditions in patients of the two groups are compared in Table 2.

**Table 2:** Comparison of intraoperative conditions and early postoperative efficacy between the two groups (x^2^ ± s)

	Observation group	Control group	*P* value	*T/X*^2^ value
Number of patients	36	128		
Operative time (min)	144 ± 10	141 ± 11	0.101	1.647
Blood loss (ml)	63 ± 6	61 ± 7	0.247	1.161
Time to anal exhaust (h)	67 ± 7	65 ± 8	0.210	1.260
Postoperative VAS score	3.4 ± 1.1	4.5 ± 1.2	0.000	− 4.582
Postoperative complications (cases)	4	15	1.000#	
Postoperative hospital stay (days)	6.0 ± 1.1	7.2 ± 1.2	0.000	− 5.229
Treatment cost (RMB yuan)	66,823 ± 4148	60,851 ± 3698	0.322	8.330

*VAS* visual analogue scale, *RMB* Ren Min Be

^a^P < 0.05 is considered statistically significant

^b^Fisher's exact test

Patients were followed up until January 1st, 2019. The observation group had a mean follow-up time of 61 months and a mean survival time of 64 months. During the follow-up period, 18 patients had recurrence or metastasis; no recurrence in the anastomotic stoma or distal rectum was detected. In the observation group, 1-year survival, 3-year survival, and 5-year survival were 100%, 83%, and 61% respectively.

The control group had a mean follow-up time of 56 months and a mean survival time of 62 months. During the follow-up period, 55 patients had recurrence or metastasis, including 5 patients with implantation metastasis to the abdominal incision. In the control group, 1-year survival, 3-year survival, and 5-year survival were 100%, 75%, and 58% respectively. Statistical analysis suggested that there was no significant difference (*P* > 0.05) between the two groups in terms of the 3- and 5-year overall survival. Comparison of postoperative oncological outcomes between the two groups is shown in Table 3.

**Table 3:** Comparison of postoperative oncological outcomes between the two groups (x̅ ± s)

Group	Number of patients		Harvested lymph nodes	Postoperative TNM staging (cases)			1-year survival	3-year survival	5-year survival
				I	II	III			
Observation group		36	14.8	5	19	12	100%	83%	61%
Control group		128	14.1	15	77	36	100%	75%	58%
*P* value			0.062			0.730^a^		0.363^a^	0.723^a^
*T/X*^2^ value			1.880			0.630		0.827	0.126

P < 0.05 is considered statistically significant

^a^Pearson Chi-square test

## Discussion

Currently, laparoscopic surgery for colorectal cancer usually requires an auxiliary incision about 4–6 cm long on the abdominal wall, for the convenience of extraction of specimens, arrangement of stapling anvils, and colorectal anastomosis. The abdominal wall incision may inevitably cause postoperative wound pain, increase the use of painkillers, delay patient ambulation and discharge, increase the risks of incision-associated complications (such as postoperative incisional infection, intestinal adhesions, and incision site tumor implantation), and significantly undermine the advantages of minimally invasive laparoscopic surgery [15–17].

In recent years, natural orifice transluminal endoscopic surgery (NOTES) has become a new direction of minimally invasive surgery [18]. Due to imperfect surgical instruments and the lack of surgical experience, NOTES is still used in simple surgeries. Complex surgery especially radical surgery for colorectal cancer still requires sufficient data and evidence-based medical support. It will take some time for NOTES to be widely used in clinical practice. Based on the advantages of laparoscopy, the combination of laparoscopy and NOTES, such as natural orifice specimen extraction surgery (NOSES), has become a field of special interest for researchers worldwide. For example, rectal eversion-resection has been reported by many researchers [12, 13, 19]. The procedure is mainly used for patients who need anterior resection for high or middle rectal cancer. However, the surgery requires rectal passage through a narrow enteric cavity, which often makes the passage difficult; mechanical compression during the eversion process also easily leads to tumor cell detachment or rupture, resulting in tumor metastasis to the intraperitoneal cavity.

To overcome the shortcomings of the above methods, a modified technique of transanal specimen extraction was used in our center. Namely, after the rectum was transected with a linear cutter stapler, the stump of the distal rectum was incised with an ultrasonic scalpel, and a sheath-shaped sterile bag was inserted into the pelvic cavity through the anus for placing the proximal intestinal segment into the bag. After the bag opening was tightened, the tumor-containing proximal colonic segment and the sterile bag were synchronously pulled out of the anus via the distal rectum. This technique meets the development trend of minimally invasive surgery, ensures aseptic conditions, and fufills the principle of no-neoplasma-touch-technique.

This technique has the following advantages: (1) compared with rectal eversion resection, it is easier to pull out a colonic segment transanally using the technique. (2) No auxiliary incision on the abdominal wall is required, which means that the procedure is truly minimally invasive and “scarless”. (3) The technique ensures aseptic and tumor-free conditions for tumor surgery. ① Before a colonic segment is pulled out, the tumor-containing proximal colonic segment is ligated to block blood flow and prevent hematogenous spread caused by tumor compression when the colonic segment is pulled out; ② During the entire pull-out process, the colonic segment is completely isolated in a sterile bag, which prevents detachment of tumor cells and implantation metastasis to the distal rectum; ③ Due to in vitro resection of the tumor, adequate and safe surgical margins can be easily determined under direct vision. During the follow-up period, no recurrence in the anastomotic site or distal rectum was found, suggesting that the modified technique of transanal specimen extraction is capable of isolating the tumor-containing colonic segments effectively.

## Key points for success

To apply this technique, the following points should be considered: (1) Before using this technique, accurate assessment is required (including determination of tumor sizes, tumor stages, length of the sigmoid colon, and capacity of the distal rectal cavity). (2) The aseptic principle in surgery should be followed. The proximal colonic segment is not opened in the intraperitoneal cavity; before opening the distal intestinal segment, the distal rectum is irrigated thoroughly with a diluted povidone-iodine solution to prevent infection caused by contamination of the abdominal cavity. (3) Before a colonic segment is pulled out, anal dilation is required for the convenience of taking out the tumor easily. If the anal opening is very tight, it would cause incarceration of the colonic segment when it is pulled out; thus, resulting in postoperative colonic vasospasm and further affecting the anastomotic blood flow and healing. (4) To pull out the proximal colonic segment, a pair of toothed oval forceps should be placed in the sterile bag and it should be used to clamp the stump of the colonic segment and pull out both the colonic segment and the bag synchronously. Pulling out the sterile bag only should not be allowed, for fear of specimen agglomeration that would make specimen extraction difficult. (5) There must be some resistance when a tumor is pulled through a rectal stump. Violent pulling should not be allowed. The rectum should be simply dilated with fingers via the anus to assist in specimen extraction and prevent tearing of the rectal stump. (6) If a specimen is too large to pass through the distal rectum, an auxiliary abdominal incision should be made in a timely manner for specimen extraction. An ultrasonic scalpel should not be used to cut apart a colonic segment and its mesentery, due to fear of iatrogenic spread of tumor cells.

The modified technique transanal specimen extraction can be used in patients with the following indications: the transverse diameter of a colonic segment with the lower sigmoid colon or upper rectal tumor should be less than 5 cm. The technique is not suitable for patients with an excessively large tumor or hypertrophic mesorectum, due to fear of rupture of the distal rectum when the proximal colonic segment is pulled out. The technique is not suitable for patients if their sigmoid colon is too short, because insufficient length of a colonic segment would make it difficult to be pull it out. The technique is not applicable to middle and lower rectal cancer, because secondary resection of the rectum is required, namely, the resection length of the distal rectum is 2 cm more than that in anterior resection for transabdominal specimen extraction, possibly resulting in lower anastomosis and relevant complications.

There were no significant differences (P > 0.05) between the two groups in terms of the operative time, and intraoperative blood loss. However, the observation group showed a significantly lower mean postoperative VAS pain score than the control group (P < 0.05), and a shorter mean postoperative hospital stay than the control group (P < 0.05), thus, showing the possible advantages of the modified technique in minimally invasive surgery. Follow-up results showed that, there were no significant differences between the two groups in terms of the 1, 3 and 5-year survival rates, thus, suggesting that the modified technique of transanal specimen extraction is reliable for antitumor treatment.

## Conclusion

Transanal sampling is one of the manifestations of NOSES, which represents the latest trend of minimally invasive technology. The modified technique of transanal specimen extraction in laparoscopic anterior rectal resection for upper rectal or lower sigmoid colon cancer is feasible and safe. Characterized by minimal trauma, rapid recovery, and fewer complications, the technique offers the benefits of minimally invasive surgery. Long-term follow-up also showed satisfactory oncological outcomes. However, our results are only based on a retrospective analysis and it is difficult for a retrospective study to achieve complete comparability between the two groups. Prospective controlled studies with a larger sample size are necessary to gain more experience.

## Data Availability

All data included in this study are available upon request from the corresponding author.

## Abbreviations

ISR: Intersphincteric resection
MRI: Magnetic resonance imaging
CT: Computed tomography
SD: Standard deviation
BMI: Body mass index
ASA: American anesthesiologist classification
VAS: Visual analogue scale
UICC: Union for International Cancer Control
TNM: Tumor, node and metastasis
NOTES: Natural orifice transluminal endoscopic surgery
NOSES: Natural orifice specimen extraction surgery
